# Probing the *Xenopus laevis* inner ear transcriptome for biological function

**DOI:** 10.1186/1471-2164-13-225

**Published:** 2012-06-08

**Authors:** TuShun R Powers, Selene M Virk, Casilda Trujillo-Provencio, Elba E Serrano

**Affiliations:** 1 Biology Department, New Mexico State University, Las Cruces, USA

**Keywords:** Amphibian, Auditory, Deafness, Hearing, Microarray, Organ, Vestibular

## Abstract

****Background**:**

The senses of hearing and balance depend upon mechanoreception, a process that originates in the inner ear and shares features across species. Amphibians have been widely used for physiological studies of mechanotransduction by sensory hair cells. In contrast, much less is known of the genetic basis of auditory and vestibular function in this class of animals. Among amphibians, the genus *Xenopus* is a well-characterized genetic and developmental model that offers unique opportunities for inner ear research because of the amphibian capacity for tissue and organ regeneration. For these reasons, we implemented a functional genomics approach as a means to undertake a large-scale analysis of the *Xenopus laevis* inner ear transcriptome through microarray analysis.

****Results**:**

Microarray analysis uncovered genes within the *X. laevis* inner ear transcriptome associated with inner ear function and impairment in other organisms, thereby supporting the inclusion of *Xenopus* in cross-species genetic studies of the inner ear. The use of gene categories (inner ear tissue; deafness; ion channels; ion transporters; transcription factors) facilitated the assignment of functional significance to probe set identifiers. We enhanced the biological relevance of our microarray data by using a variety of curation approaches to increase the annotation of the *Affymetrix* GeneChip® *Xenopus laevis* Genome array. In addition, annotation analysis revealed the prevalence of inner ear transcripts represented by probe set identifiers that lack functional characterization.

****Conclusions**:**

We identified an abundance of targets for genetic analysis of auditory and vestibular function. The orthologues to human genes with known inner ear function and the highly expressed transcripts that lack annotation are particularly interesting candidates for future analyses. We used informatics approaches to impart biologically relevant information to the *Xenopus* inner ear transcriptome, thereby addressing the impediment imposed by insufficient gene annotation. These findings heighten the relevance of *Xenopus* as a model organism for genetic investigations of inner ear organogenesis, morphogenesis, and regeneration.

## **Background**

Hearing and balance are essential for animal communication and locomotion. Auditory and vestibular disorders limit the perception of sound and spatial orientation. In humans, such disorders detract from the quality of life through the impact they have on other activities, such as social interaction, education, and mobility. Diminished senses of hearing and balance frequently result from abnormalities in the organs of the inner ear. The World Health Organization (WHO) estimates that hearing impairment and deafness impact over 278 million people, making sensorineural hearing loss a prevalent sensory disorder in humans worldwide [1]. The incidence of vestibular disorders is more difficult to determine because of diagnostic challenges. Balance disabilities may reflect the abundance of vestibular disorders such as Ménière’s disease, labyrinthitis, benign paroxysmal positional vertigo (BPPV) and vestibular neuritis [2,3]. In the United States, the incidence of Ménière’s disease is estimated to increase by about 45,000 persons each year [4]. Excessive ambient noise, aging populations, exposure to ototoxic drugs, and the inheritance of genetic mutations are believed to contribute to the prevalence of hearing and balance disorders. Understanding how environmental and genetic factors directly impact the function of the inner ear is therefore critical to the treatment and alleviation of auditory and vestibular problems [5,6].

The senses of hearing and balance depend on the conversion of mechanical stimuli into neural signals by the auditory and vestibular endorgans of the inner ear [7]. The endorgans contain sensory epithelia that comprise mechanoreceptor sensory hair cells and supporting cells [7,8]. Damage to endorgan tissue, such as injury to hair cells and the eighth cranial nerve, can cause sensorineural hearing loss and vestibular disorders [7,8]. Current understanding of inner ear biology stems from research that has focused on genetics, determining molecular elements required for hair cell function and regeneration, endorgan development, and identifying ototoxic factors and molecular targets for therapeutic treatments [5,9].

Although the inner ear endorgans of mammals and non-mammals are morphologically distinct, mechanosensory hair cells share physiological and structural similarities across species [8]. Cross-species comparisons of mammals (mouse, human, rat, chinchilla, guinea pig), reptiles (turtles), birds, amphibians, and fish have collectively defined our current understanding of the processes of hair cell mechanotransduction, regeneration and transdifferentiation [10-16]. Genetic analysis has provided insight into the hereditary basis of deafness in humans and mice [17-21]. Large-scale transcriptome analysis tools such as cDNA libraries and microarrays have been used to identify inner ear genes in human, mouse, chicken, rat, and zebrafish [22-28]. Outcomes of these investigations have established cross-species similarities in the genetic profile of the inner ear.

Physiological and anatomical investigations of the class *Amphibia* have been seminal to our understanding of the cellular basis of auditory and vestibular processing. In particular, studies on the process of mechanotransduction in amphibian hair cells have formulated the framework for elucidating the biophysical details of hair cell mechanoreception [29-31]. Moreover, amphibians (along with birds and fish) have been shown to regenerate or transdifferentiate hair cells after trauma and therefore are a useful model for inner ear research [13,14,32]. Outcomes of experiments with amphibian genera such as *Rana* (*R. catesbeiana,* bullfrog; *R. pipiens*, leopard frog; *R. temporaria*, grass frog), *Hyla* (*H. cinerea,* green tree frog), and the African clawed frog*, Xenopus (X. laevis; X. tropicalis),* have contributed to our knowledge of peripheral sound reception and otoacoustic emissions [33,34] as well as sensory endorgan development [35-38].

In contrast to the emphasis on amphibians as model organisms for investigations of hair cell electrophysiology and mechanotransduction, amphibians have been underutilized as models for analysis of global gene expression in the inner ear. This omission may be partially attributed to the novelty of transcriptional profiling and similar large-scale genetic analyses as tools for uncovering inner ear function in any species [23-28]. Although inner ear genes have been characterized individually in amphibians and other species [36,39-41], large-scale transcriptome analysis has unprecedented potential to significantly advance the field of inner ear genetics [23,27,28,42].

Among amphibians, the genus *Xenopus* offers unique opportunities for genetic investigations of inner ear structure and function due to the availability of a sequenced genome [43], and the thorough characterization of developmental stages [44,45]. Furthermore, *Xenopus* is well suited to genetic analysis because methods that enable the production of thousands of transgenic embryos are well established [46,47]. Online resources specific to *Xenopus*, such as XenDB and Xenbase, facilitate cross-species genetic analysis [48,49]. In addition, transcriptional profiling with microarrays has been used for large-scale analysis of *Xenopus* gene expression to investigate early embryonic development, non-inner ear organ specific expression, and limb regeneration [50-54]. Genetic findings from such large-scale approaches can be contextualized by the aforementioned physiological studies of amphibian hair cell function.

Transcriptional profiling of *Xenopus* inner ear endorgans can potentially identify gene families and expression patterns that typify functional inner ear tissue. To this end, we used microarray analysis to ascertain the genetic basis of *Xenopus* auditory and vestibular sensation. We profiled RNA isolated from the inner ears of juvenile animals, a developmental age where all anatomical structures are fully formed, and animals are in the initial stages of postmetamorphic life [44]. The *Affymetrix* GeneChip® *Xenopus laevis* Genome array version 1 (*X. laevis* GeneChip®) was used for the identification of key molecular components of the *X. laevis* inner ear.

The unknown biological function of many *X. laevis* probe set identifiers (Xl-PSIDs) on the GeneChip®, a drawback that stems in part from the unsequenced *X. laevis* genome, prompted our use of extensive manual curation efforts to augment the functional significance of the array data. In order to relate prior knowledge of genes with predicted inner ear function to the *X. laevis* inner ear transcriptome, we focused our *X. laevis* GeneChip® annotation efforts on five inner ear gene categories: genes that encode ion channels (IC), ion transporters (IT), and transcription factors (pTF); genes found in inner ear tissue (IET); and genes with mutations that cause deafness (DF). Sequence similarity mapping, semantic keyword querying and the *XenEnhance* relational database [55] enabled linkage of the more informative official gene symbols from the HUGO Gene Nomenclature Committee (HGNC, [56]) to a subset of Xl-PSIDs on the *X. laevis* GeneChip® [54,55]. Throughout this paper we use the HGNC nomenclature to refer to genes of interest.

We approached our analysis of the *X. laevis* inner ear transcriptome by examining the intensity levels and functional classification of Xl-PSIDs. As expected, Xl-PSIDs with high intensity values corresponded to genes that are predominantly involved in housekeeping and maintenance functions common to many cell and tissue types. Intensity analysis also highlighted the prevalence of Xl-PSIDs with no known annotation or protein counterpart. Our comparison of the inner ear gene categories revealed that the transcription factor gene category was characterized by the lowest Xl-PSID intensity value distribution of all five categories.

Our manual curation efforts enabled us to evaluate whether genes associated with inner ear function in other organisms (human, rat, mouse, and chicken) were potentially represented either in the *X. tropicalis* genome or on the *X. laevis* GeneChip®. Sequence similarity alignments revealed that the majority of HGNC protein sequences from the inner ear gene categories have counterparts in the *X. tropicalis* genome and on the *X. laevis* GeneChip®. Taken together, the results of our transcriptome analysis demonstrate that *Xenopus* is a viable model organism for auditory and vestibular research, and support the inclusion of *Xenopus* in cross-species comparisons. Our results also have uncovered potential gene targets that, through transgenic approaches, have the potential to reveal genetic elements of inner ear function and dysfunction.

## **Results**

### **Data normalization and distribution**

**Figure 1:**
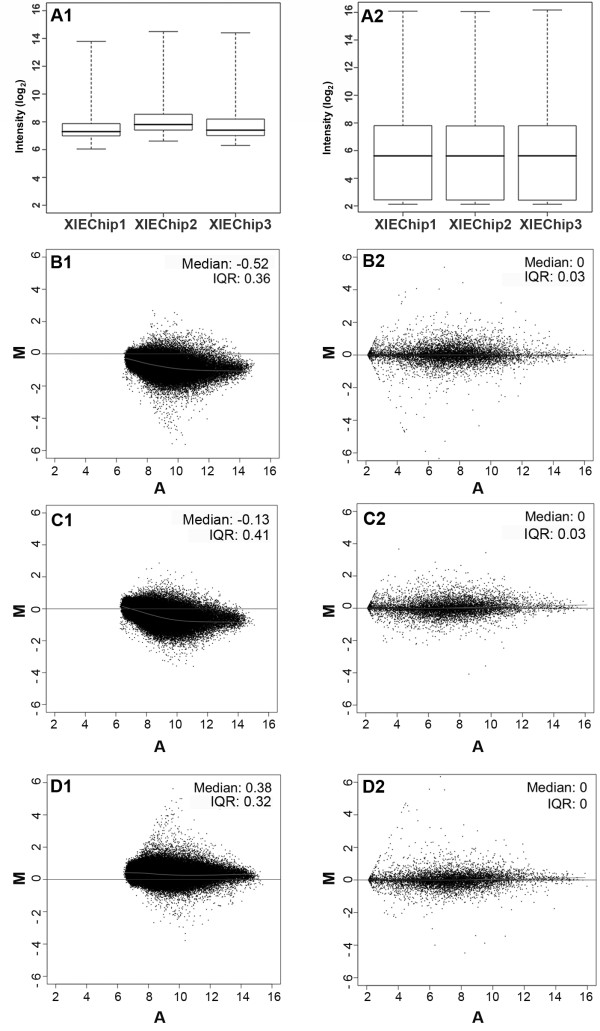
** Normalization of *X. laevis* inner ear tissue (XIE) microarray data.A.** Box plots of pre-normalized (A1) and GCRMA normalized (A2) Xl-PSID intensity data from three replicate XIE chips. **B-D.** MvA plots for pre-normalized (1) and GCRMA normalized (2) Xl-PSID intensity values representing the difference between chips XIE1-XIE2 (B), XIE1-XIE3 (C), and XIE2-XIE3 (D). Y axis (M, minus), differences in intensity for any given Xl-PSID from the two arrays. X axis (A, average), average intensity for a given Xl-PSID on the two arrays. Median and average IQR values for the Xl-PSID intensities are given on each plot.

The microarray CEL file raw data were preprocessed using the GeneChip robust multichip analysis (GCRMA) summarization method. The distribution of Xl-PSID intensity values for the normalized data ranged from 2.12-16.01 (see Additional file 1). Box plots of triplicate *X. laevis* inner ear (XIE) arrays illustrate the similarity between replicates for both pre- and post-normalized data (Figure 1A1, 1A2). MvA plots demonstrate the benefit of normalization and illustrate the same trend between the replicates as seen with box plots (Figure 1B1-1D2). As with the box plots, MvA plots of pre-normalized data (Figure 1B1, 1 C1, and 1D1) showed an asymmetrical distribution of data and greater inter-chip variation than normalized data (Figure 1B2, 1 C2, and 1D2). The interquartile range (IQR) values were very low for normalized data (Figure 1B2, 0.03; 1 C2, 0.03; 1D2, 0) and much less than for pre-normalized data (Figure 1B1, 0.36; 1 C1, 0.41; 1D1, 0.32).

Similarity among the replicate arrays was demonstrated by the analysis of the inter-chip and intra-chip averages and standard deviations (SDs) for normalized Xl-PSID intensity values. The average Xl-PSID intensity values for the individual chips were almost identical; the inter-chip SD was 2.3% of the inter-chip average Xl-PSID intensity value of 5.62. The individual intra-chip SDs also were of comparable magnitude and ranged from 2.94 to 2.97 (Table 1). In our analysis of the *X. laevis* inner ear transcriptome we excluded the control Xl-PSIDs (*n* = 120), a procedure that raised the inter-chip average Xl-PSID intensity from 5.62 to 5.63 (Table 1).

**Table 1:** Intra-chip and inter-chip average Xl-PSID intensity values (a.u.)

	**Intra-chip averages**			**Inter-chip averages**
	**XIE1**	**XIE2**	**XIE3**	**XIE**
	**Xl-PSID intensity**	**Xl-PSID intensity**	**Xl-PSID intensity**	**Xl-PSID intensity**
**All Xl-PSIDs** (*n* = 15611)	5.62 + 2.97	5.61 + 2.96	5.61 + 2.94	5.62 + 0.13
**Control Xl-PSIDs** (*n* = 120)	4.10 + 3.83	4.06 + 3.78	4.05 + 3.78	4.07 + 0.05
**Experimental** **Xl-PSIDs** (*n* = 15491)	5.64 + 2.96	5.63 + 2.95	5.62 + 2.93	5.63 + 0.13
**Xl-PSIDs with** **“P”/”M” GCOS calls** **(***n* = 12177)	6.55 + 2.67	6.54 + 2.66	6.54 + 2.65	6.54 + 0.17
**Xl-PSIDs with “A” GCOS calls in all 3 XIE chips** **(***n *= 3314)	2.27 + 0.41	2.26 + 0.40	2.26 + 0.39	2.67 + 0.01

GCRMA intra-chip averages are the calculated average intensity of the normalized Xl-PSIDs on a given chip (XIE1, XIE2, or XIE3; see Additional file 1). GCRMA inter-chip averages are computed as the average intensity of all average Xl-PSID intensity values for all three chips. Data are presented as average + SD.

### **Genes that correspond to Xl-PSID consensus sequences can be amplified with RT-PCR from*X. laevis*inner ear RNA**

**Figure 2:**
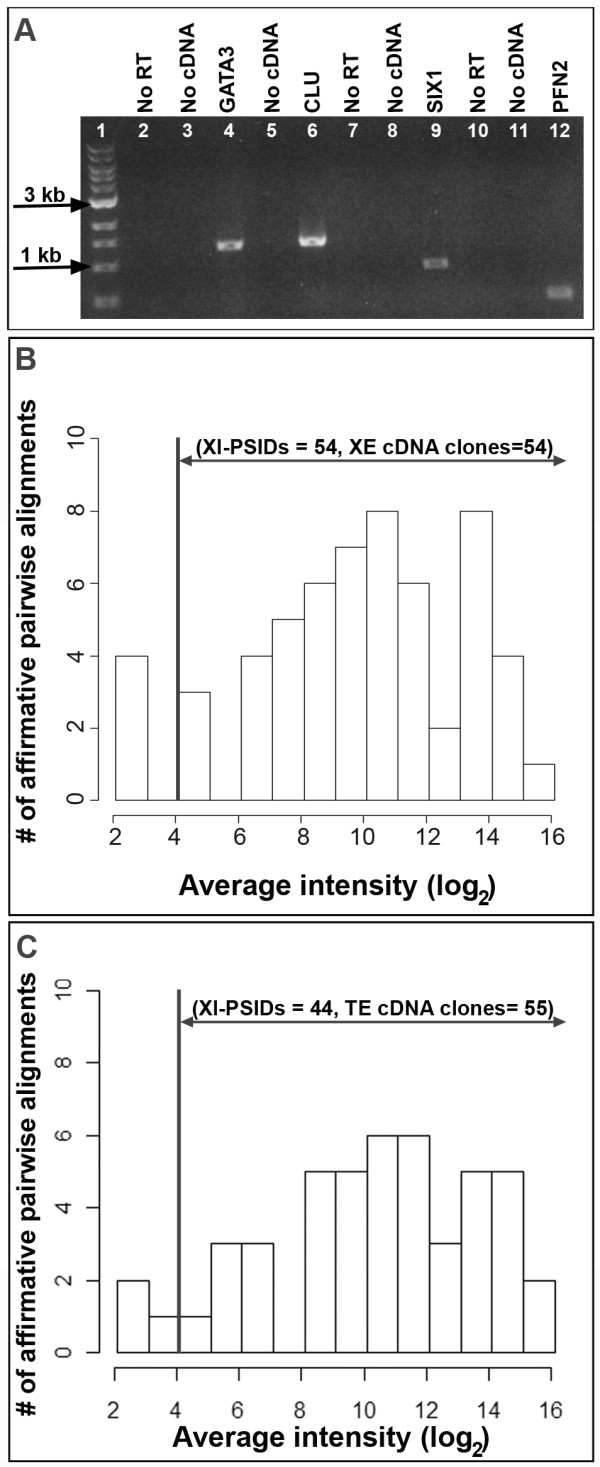
** RT-PCR analysis with *Xenopus* inner ear RNA.A**. Electrophoresis gel of PCR products from RT-PCR reactions with template inner ear RNA. Lane 1: New England BioLabs 1 kb DNA ladder; Lane 2: No RT control with *gata3* primers; Lane 3: No cDNA control with *gata3* primers; Lane 4: *gata3* amplified product; Lane 5: No cDNA control with *clu* primers; Lane 6: *clu* amplified product; Lane 7: No RT control with *six1* primers; Lane 8: No cDNA control with *six1* primers; Lane 9: *six1* amplified product; Lane 10: No RT control with *pfn2* primers; Lane 11: No cDNA control with *pfn2* primers; Lane 12: *pfn2* amplified product. **B**-**C**. Histograms of the average intensities of 105 Xl-PSID consensus sequences that formed affirmative pairwise alignments (BLASTN) with *X. laevis* (B, XE, *n* = 58) and *X. tropicalis* (C, TE, *n* = 58) inner ear cDNA library clones. Vertical line indicates an intensity value of four.

Primers were designed against consensus sequences for eight Xl-PSIDs with varied intensity levels (see Methods): gene name, gene symbol (average intensity ± SD), GATA binding protein 3, *gata3* (6.85 ± 0); clusterin, *clu* (14.94 ± 0.02); profilin 2, *pfn2* (12.72 ± 0.09); SIX homeobox 1, *six1* (10.70 ± 0.07); matrilin 2, *matn2* (8.14 ± 0.23); peripheral myelin protein 22, *pmp22* (14.29 ± 0.34); chloride channel, voltage-sensitive Ka, *clcnkb* (8.98 ± 0.47); and sodium channel, non-voltage-gated 1, beta subunit, *scnn1b* (9.69 ± 0.10). RT-PCR products were obtained with all eight primer pairs. Figure 2A shows the amplification bands for *gata3*, *clu*, *pfn2*, and *six1*. DNA sequencing confirmed the predicted identity of all RT-PCR products. The eight consensus sequences corresponded to genes associated with ion transport, the extracellular matrix, hearing impairment, and deafness.

### **Sequence similarity between* Xenopus* inner ear cDNA library clones and Xl-PSID consensus sequences**

The BLASTN algorithm was used to find homology between 197 clones from two *Xenopus* inner ear cDNA libraries (*X. laevis*, XE, *n* = 96; *X. tropicalis*, TE, *n* = 101; [57]) and Xl-PSID consensus sequences (refer to Methods). The number of BLASTN derived pairwise alignments in each similarity group (high, H; moderate, M; weak, W; and low, L) based on expect values (e-values) are shown in Table 2. We noted that in some instances more than one cDNA library clone aligned with the same Xl-PSID. Consequently, the number of cDNA/Xl-PSID affirmative pairwise alignments (*n* = 116) was greater than the number of target Xl-PSIDs (*n* = 105) on the *X. laevis* GeneChip®. The five Xl-PSIDs with multiple cDNA clone alignments represent hemoglobin, gamma G (*hbg2-a*); ferritin light chain (*ftl*); ribosomal protein S12 (*rps12a*); an unknown sequence; and cytochrome c oxidase subunit Va (*cox5a*). 

**Table 2:** **Pairwise alignments of* Xenopus* cDNA clones and Xl-PSID consensus sequences: Similarity groupings by e-value**

**Similarity group**	**Number of XE/Xl-PSID pairwise alignments**	**Number of TE/Xl-PSID pairwise alignments**
**High (e = 0-10**^**-100**^**)**	45	31
**Moderate (e = 10**^**-99**^**-10**^**-50**^**)**	4	17
**Weak (e = 10**^**-49**^**-10**^**-15**^**)**	9	10
**Low (e > 10**^**-14**^**)**	38	43
**Affirmative pairwise alignments (H, M, W)**	58	58 (47 unique Xl-PSIDs)

Xl-PSIDs were aligned to *Xenopus laevis* (XE) and *Xenopus tropicalis* (TE) inner ear library clone sequences using the BLASTN algorithm. Pairwise alignments were sorted into similarity groups based on e-value. Xl-PSIDs with multiple pairwise alignments to cDNA library clones were counted once. Four Xl-PSIDs aligned to two TE cDNA clones, and one Xl-PSID aligned to eight TE cDNA clones.

When we analyzed the intensity distribution of the 105 Xl-PSIDs with affirmative pairwise alignments with 116 inner ear cDNA clones, we noted that 93.3% of the cDNA clones mapped to Xl-PSIDs with average intensity levels greater than four (Figure 2B, 2C). The seven cDNA clones that mapped to Xl-PSIDs with average intensity values under four represented unknown sequences, the BMP4 gene, spondin 2 (extracellular matrix protein), and prolyl 4-hydroxylase, beta polypeptide. We also found that more than 98% of all experimental Xl-PSIDs that were designated “*A*” by the *Affymetrix* GCOS software (see Methods) had average intensity levels below four (*n* = 3269, see Additional file 1). Based on these observations, we expect that an Xl-PSID intensity value greater than or equal to four is likely to represent an expressed inner ear sequence.

### **Xl-PSID intensity analysis with decile groupings and functional characterization**

As a prelude to functional analysis, we rank ordered the Xl-PSIDs (*n* = 12,177; Table 1) based on their average intensity values. The ten Xl-PSIDs with the highest intensity values were: hemoglobin, gamma A, *hbg1*; ribosomal protein S27, *rps27*; ferritin (heavy polypeptide 1 a), *fth1*; ubiquitin B, *ubb*; ribosomal protein S13, *rps13*; solute carrier family 11 (proton-coupled divalent metal ion transporters), member 2, *slc11a2*; ribosomal protein S20, *rps20*; 1 unknown sequence; ribosomal protein S14, *rps14*; and hypothetical protein MGC114621/ribosomal protein (large, P1), *rplp1*.

We partitioned the Xl-PSIDs into deciles by two methods, equal number of Xl-PSIDs (Table 3A, equal tally deciles) and equal range of average intensity values (Table 3B, equal intensity deciles). For equal tally deciles, the variation of Xl-PSID average intensity values were low and comparable (~0.59-1.31) for all the deciles except for the 10^th^ (6.16). In contrast to equal tally deciles, 40 (0.33%) of the Xl-PSIDs in the equal intensity deciles were grouped in the 10^th^ decile (14.62-16.01).

**Table 3:** Xl-PSID distribution in equal tally and equal intensity deciles

**3A**				**3B**			
**Equal tally decile**	**Difference in intensity value (high-low)**	**Xl-PSID counts**	**Orphan Xl-PSIDs**	**Equal intensity decile**	**Intensity value range**	**Xl-PSID counts**	**Orphan Xl-PSIDs**
10	6.16	1218	159	10	1.39	40	1
9	1.14	1218	266	9	1.39	118	1
8	0.78	1218	351	8	1.39	175	17
7	0.68	1218	393	7	1.39	522	74
6	0.62	1218	414	6	1.39	1186	227
5	0.72	1218	436	5	1.39	2134	610
4	0.81	1218	459	4	1.39	2608	893
3	1.06	1217	457	3	1.36	2034	762
2	1.31	1217	500	2	1.36	1454	566
1	0.59	1217	516	1	1.36	1906	800

Xl-PSIDs were divided into decile groupings either by number (A) or intensity (B); 10th decile corresponds to the highest intensity levels. In the equal tally distribution (A), each decile comprises an equal number (*n* = 1217/1218) of Xl-PSIDs. In the equal intensity distribution (B), each decile comprises Xl-PSIDs within an equal intensity range (*n* = 1.36/1.39 a.u.). The number of Xl-PSIDs without annotation after DAVID queries is listed in the Orphan Xl-PSID column.

We focused our functional analysis on the 10^th^ deciles, which comprise Xl-PSIDs with the highest average intensity values in both instances. The Database for Annotation, Visualization and Integrated Discovery (DAVID, [58,59] was used to classify and cluster Xl-PSIDs with Gene Ontology (GO), KEGG and SP-PIR terms.

As shown on Table 4A the most common functional annotation for the 10th equal tally decile was the GO term “*cellular processes*” (30%). DAVID analysis also classified the top 10% of Xl-PSIDs into other annotation categories, including “*biosynthetic processes”*, “*gene expression”*, “*translation*”, “*non-membrane-bounded organelle*”, and “*structural molecule activity*”. The most common functional annotations for the 10th equal intensity decile (Table 4B) were: “*non-membrane-bounded organelle*”, “*intracellular non-membrane-bounded organelle*”, and “*translation*”. We observed that some Xl-PSIDs were clustered in multiple functional categories. Moreover, DAVID reported an “orphan” (i.e. no annotation retrieved [60]) status for 13.1% of the Xl-PSIDs in the 10^th^ equal tally decile and 2.5% of the Xl-PSIDs in the 10^th^ equal intensity decile (Table 3). We also noted a similar trend in both decile groupings; the number of orphan Xl-PSIDs within a decile decreased as the intensity values of their decile increased. In comparison to DAVID, 20.6% (251/1218) of the Xl-PSIDs in the 10^th^ equal tally decile and 10% of the Xl-PSIDs in the 10^th^ equal intensity decile (4/40) were without gene annotation based on the annotation file provided by the vendor (Xenopus_laevis.na32.annot.csv, [61]). 

**Table 4:** **DAVID functional clustering of Xl-PSIDs in the 10**^**th**^** equal tally and 10**^**th**^** equal intensity deciles**

**4A 10th Equal tally decile**		
**GO terms**		**Number of DAVID IDs**
	cellular process	366
	metabolic process	322
	primary metabolic process	287
	cellular metabolic process	278
	macromolecule metabolic process	204
	macromolecular complex	198
	cellular macromolecule metabolic process	190
	cytoplasmic part	190
	biosynthetic process	176
	protein metabolic process	175
	cellular biosynthetic process	171
	cellular protein metabolic process	161
	gene expression	138
	intracellular non-membrane-bounded organelle	137
	non-membrane-bounded organelle	137
	cellular macromolecule biosynthetic process	129
	macromolecule biosynthetic process	129
	structural molecule activity	118
	translation	112
	ribonucleoprotein complex	93
	structural constituent of ribosome	82
	Ribosome	82
**SP_PIR_Keywords**		
	ribonucleoprotein	55
	ribosomal protein	54
**KEGG**		
	Ribosome	73
**4B 10**^**th**^**Equal intensity decile**		
**GO terms**		**Number of DAVID IDs**
	translation	26
	non-membrane-bounded organelle	26
	Intracellular non-membrane-bounded organelle	26
	structural constituent of ribosome	25
	Ribosome	25
	structural molecule activity	25
	ribonucleoprotein complex	25
**SP_PIR_Keywords**		
	ribosomal protein	16
	ribonucleoprotein	15
**KEGG**		
	Ribosome	22

The functional clusters with the highest DAVID enrichment scores are shown for Xl-PSIDs in the 10th equal tally (A) and the 10th equal intensity (B) decile. DAVID assigned 905 IDs to the 10^th^ equal tally decile, resulting in 121 annotation clusters. DAVID assigned 35 IDs to the 10^th^ equal intensity decile, resulting in 3 annotation clusters.

### **Assigning inner ear gene categories to Xl-PSIDs**

**Figure 3:**
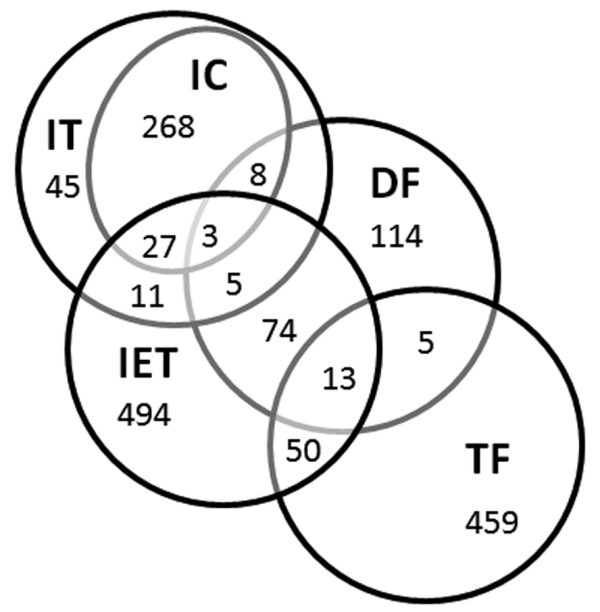
** Venn diagram of the five inner ear gene categories.** Venn diagram showing the number and overlap of HGNC or gene symbols within the five inner ear gene categories (see Additional file 2). The total number of symbols in each inner ear gene category are: 680 (IET); 222 (DF); 306 (IC); 367 (IT); and 527 (pTF). One gene symbol, *NR3C1*, was included in three gene lists (IET, 681; IT, 368; pTF, 528) and excluded from the diagram.

As a prelude to analyzing the inner ear transcriptome, we identified Xl-PSIDs on the *X. laevis* GeneChip® with a probable role in the maintenance and function of auditory and vestibular inner ear endorgans. We selected five gene categories for intensity analysis: inner ear tissue (IET), deafness (DF), ion channels (IC), ion transport (IT), and transcription factors (pTF). The Venn diagram in Figure 3 shows the overlap between the five inner ear gene categories (see Additional file 2). Several approaches were used to assign these gene categories to Xl-PSIDs (Table 5, see Methods).

**Table 5:** Summary of gene compilation and analysis methods

**Gene group**	**Xl-PSIDs**	**HGNC symbols**	**Method of compilation**	Sequence similarity mapping	**DAVID analysis**
**IET**	453	594*	Scientific literature [22-27,42,85]	All	Yes
**DF**	139	157*	Keyword query (OMIM database)	All	No
**IC**	74	210*	Scientific literature [66,88-90]	All	No
**IT**	180	130	Keyword query (*Affymetrix* annotation file) and scientific literature	Subset (IC)	No
**pTF**	795	790*	Keyword query (NetAffx™ analysis center and Xenbase)	----	Yes
**10**^**th**^**equal tally decile**	1218	----	Rank ordered top 10% based on number of Xl-PSIDs	----	Yes
**10**^**th**^**equal intensity decile**	40	----	Rank ordered based on intensity value range	----	Yes

The number of Xl-PSIDs in all inner ear gene categories (IET, DF, IC, IT, pTF) and in the 10^th^ deciles are reported together with compilation and analysis methods for each gene group. The table reports the number of symbols for HGNC proteins that formed affirmative pairwise alignments with Xl-PSID consensus sequences through sequence similarity mapping (TBLASTN). *HGNC symbols were linked to more than one Xl-PSID, resulting in a number that is higher than the number Xl-PSIDs.

### **Mapping IET, DF and IC inner ear gene categories to Xl-PSIDs**

To assess the utility of the *X. laevis* GeneChip® in inner ear array studies, we evaluated whether genes associated with inner ear function in *Xenopus* and other organisms (e.g. human, rat, mouse, and chicken) were arrayed on the chip. To this end, we used sequence similarity mapping with the TBLASTN algorithm to determine whether HGNC human protein sequences from the IET, DF, and IC gene lists aligned with Xl-PSID consensus sequences (see Methods). The top BLAST pairwise alignment was used to assign putative function to the Xl-PSID consensus sequence (see Methods). HGNC human protein sequences (*n* = 855) formed affirmative pairwise alignment with 577 Xl-PSID consensus sequences. We noted that in some instances a single Xl-PSID aligned with multiple human protein sequences (19% of IET/Xl-PSIDs, 11% of DF/Xl-PSIDs, and 51% of IC/Xl-PSIDs).

### **Xl-PSID intensity analysis of inner ear gene categories**

**Figure 4:**
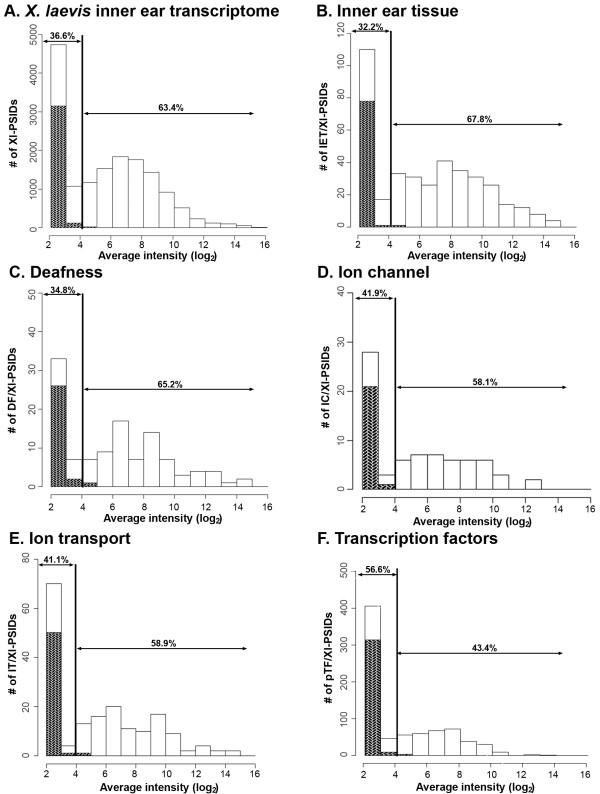
**Histograms of Xl-PSID intensity values.A**. Distribution of average Xl-PSID intensities for all experimental Xl-PSIDs (*n* =15, 491). Shaded areas are Xl-PSIDs with GCOS absent calls in all three replicates (*n* = 3, 314). **B**-**F**. Distribution of average intensities for Xl-PSIDs in the five gene categories: B, inner ear tissue (IET/Xl-PSIDs, *n* = 453); C, deafness (DF/Xl-PSIDs, *n* = 139); D, ion channel (IC/Xl-PSIDs, *n* = 74); E, ion transport (IT/Xl-PSIDs, *n* = 180); F, transcription factors (pTF/Xl-PSIDs, *n* = 795). Shaded areas are Xl-PSIDs with GCOS absent calls in all three replicates (B, IET/Xl-PSIDs, *n* = 92; C, DF/Xl-PSIDs, *n* = 33; D, IC/Xl-PSID, *n* = 22; E, IT/Xl-PSIDs, *n* = 52; F, pTF/Xl-PSIDs, *n* = 328). Vertical line separates the percentage of Xl-PSIDs intensities above and below four.

As shown in Figure 4A, the histogram of average intensity values for all experimental Xl-PSIDs on the microarray was characterized by an asymmetrical left-skewed distribution. Since approximately 63.4% of Xl-PSIDs were scored with average intensity levels above four (Figure 4A), we estimated that two-thirds of the *X. laevis* GeneChip® could be used to detect *Xenopus* inner ear transcripts. The histograms of average intensity values for each of the five gene categories also showed an asymmetrical left-skewed distribution. The majority of Xl-PSIDs were scored with average intensity levels greater than or equal to “four” in all gene categories except “transcription factor” (Figure 4B-4F).

### **Inner ear tissue genes**

**Figure 5:**
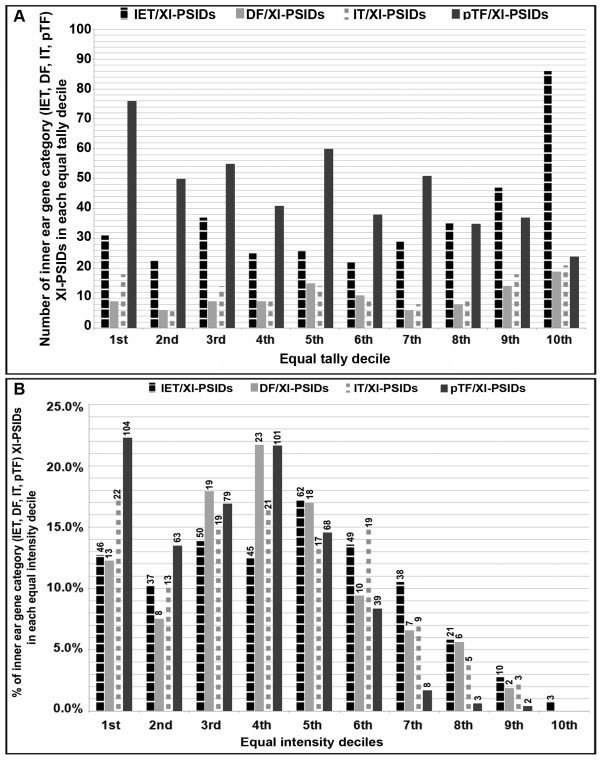
** Decile analysis of inner ear gene category Xl-PSIDs.** Bar graphs show the distribution Xl-PSIDs in each equal tally (**A**, number) or equal intensity (**B**, percentage) decile for IET/Xl-PSIDs (*n* = 361); DF/Xl-PSIDs (*n* = 106); IT/Xl-PSIDs (*n* = 128); pTF/Xl-PSIDs (*n* = 467). Note that IT/Xl-PSIDs includes both IC and IT genes.

Approximately 87.2% of genes from the IET list were linked by affirmative pairwise alignments to Xl-PSIDs (IET/Xl-PSIDs; see Additional file 3) with intensities ranging from 2.12 to 14.94. Average intensities above four were detected from approximately 68% of IET/Xl-PSIDs (Figure 4B). We noted that about 36.8% of the IET/Xl-PSIDs were clustered in the top two equal tally deciles (9^th^ and 10^th^; Figure 5A). The range of intensities (10.01-14.94) for IET/Xl-PSIDs was greatest in the 10^th^ decile. When the IET/Xl-PSIDs were grouped into equal intensity deciles, the 5^th^ decile contained the most IET/Xl-PSIDs with intensities that ranged from 7.66-9.01 (Figure 5B). The IET genes linked to the 10 Xl-PSIDs with the highest intensities are listed in Table 6A (e.g. an apolipoprotein, subunits of ATPases, and the extracellular matrix).

**Table 6:** Top 10 Xl-PSIDs in each inner ear gene category

**6A. IET/Xl-PSID**				**6B. DF/Xl-PSID**			
**Xl-PSID**	**Average intensity**	**Rank**	**IET HGNCs**	**Xl-PSID**	**Average intensity**	**Rank**	**DF HGNCs**
Xl.10055.1.S1_at	14.94	1	CLU	Xl.2292.1.S1_at	14.29	1	PMP22
Xl.21377.1.S1_a_at	14.81	2	RPS4X	Xl.24754.1.S1_a_at	14.16	2	RPS19
Xl.23752.1.S1_x_at	14.68	3	RPS3A	Xl.8924.1.A1_at	13.04	3	GJB2
Xl.212.2.S1_a_at	14.34	4	FRZB	Xl.8851.1.S1_at	12.83	4	ITM2B
Xl.2292.1.S1_at	14.29	5	PMP22	Xl.20900.1.S1_at	12.44	5	CD151
Xl.8860.1.S1_at	14.08	6	TPT1	Xl.4138.2.S1_x_at	12.42	6	ACTB
Xl.509.1.S1_at	14.06	7	ATP1B2	Xl.606.1.S1_s_at	12.31	7	COL2A1
Xl.2617.1.S1_at	13.87	8	RPSA	Xl.26213.1.S1_at	12.13	8	COL1A1
Xl.4905.1.S1_at	13.8	9	GSTM4	Xl.2652.1.S1_at	11.77	9	PLOD3
Xl.21686.1.S1_at	13.74	10	ATP1A1	Xl.1023.1.S2_at	11.36	10	POU3F4
**6 C. IC/Xl-PSID**				**6D. IT/Xl-PSID**			
**Xl-PSID**	**Average Intensity**	**Rank**	**IC HGNCs**	**Xl-PSID**	**Average intensity**	**Rank**	**IT HGNCs**
Xl.24385.1.S1_at	12.51	1	VDAC2	Xl.3792.1.S1_x_at	14.31	1	ATP1A1
Xl.23903.1.S1_at	12.32	2	FXYD3	Xl.509.1.S1_at	14.06	2	ATP1B2
Xl.1198.1.S1_at	10.85	3	GRID1	Xl.3792.1.S1_s_at	13.26	3	ATP1A1
Xl.9482.1.A1_at	10.44	4	ABP1	Xl.8924.1.A1_at	13.04	4	GJB2
Xl.11705.1.S1_at	10.42	5	SLC25A12	Xl.6045.1.S1_at	12.54	5	ATP1B3
Xl.21929.1.S1_at	9.70	6	KCNK1	Xl.24385.1.S1_at	12.51	6	VDAC2
Xl.21035.1.S1_at	9.69	7	SCNN1B	Xl.23903.1.S1_at	12.32	7	FXYD3
Xl.6273.1.S1_at	9.68	8	VDAC1	Xl.18325.1.A1_at	12.24	8	
Xl.1407.1.S1_at	9.55	9	KCNAB2	Xl.8573.2.S1_a_at	11.67	9	ATP6V0C
Xl.17482.1.S1_at	9.53	10	GRINA	Xl.8573.2.S1_x_at	11.67	10	ATP6V0C
**6E. pTF/Xl-PSID**							
**Xl-PSID**	**Average intensity**	**Rank**	**gene symbol**				
Xl.25811.2.S1_a_at	13.55	1	*atf4*				
Xl.25811.1.S1_x_at	13.36	2	*atf4*				
Xl.3536.1.S1_x_at	12.59	3	*btf3*				
Xl.3536.2.S1_x_at	12.27	4	*btf3*				
Xl.3536.2.S1_a_at	12.23	5	*btf3*				
Xl.1023.1.S2_at	11.36	6	*pou3f4*				
Xl.3360.1.S1_a_at	10.96	7	*ilf2*				
Xl.12057.2.A1_a_at	10.78	8	*srebf1*				
Xl.4461.1.A1_at	10.76	9	*ldb1*				
Xl.683.1.S1_at	10.7	10	*six1*				

The Xl-PSIDs with the 10 highest intensity values are listed for each inner ear gene category. The corresponding HGNC symbols or gene symbols are listed for all categories. In (D), two pairs of Xl-PSIDs correspond to the same HGNC symbol and have probes derived from the same consensus sequence. One Xl-PSID in (D) did not have a HGNC symbol due to the lack of sequence similarity to a human orthologue.

### **Human deafness genes**

Approximately 71% of DF genes were linked by affirmative pairwise alignments to Xl-PSIDs (DF/Xl-PSIDs, see Additional file 4) with average intensities from 2.12-14.29. Figure 4C shows that 66.2% of DF/Xl-PSIDs had average intensities greater than four. DF/Xl-PSIDs were predominantly in the 5^th^, 9^th^, and 10^th^ equal tally deciles (Figure 5A). Whereas, when DF/Xl-PSIDs were grouped into equal intensity deciles, the distribution was mostly in the 3^rd^, 4^th^, and 5^th^ deciles (Figure 5B). The DF genes linked to the 10 Xl-PSIDs with the highest intensities are listed in Table 6B and represent various cellular functions.

### **Ion channel genes**

Approximately 69% of IC genes were linked by affirmative pairwise alignments to sequences for 74 Xl-PSIDs (IC/Xl-PSIDs, see Additional file 5) that ranged in intensity from 2.12 to 12.51. The small number of Xl-PSIDs relative to the number of genes (210) is partially due to the fact that many ion channel pore subunits aligned to the same Xl-PSID. As a group, the IC/Xl-PSIDs have lower average intensity levels than both IET/Xl-PSIDs and DF/Xl-PSIDs (Figure 4). Only 58.1% of IC/Xl-PSIDs had average intensity values greater than four (Figure 4D). The IC genes linked to Xl-PSIDs with the highest intensities were voltage-dependent anion-selective channels, glutamate receptors, and subunits from K^+^ and Na^+^ channels (Table 6C).

### **Ion transport genes**

The 180 Xl-PSIDs that represent IT and IC genes on the *X. laevis* GeneChip® (IT/Xl-PSIDs, see Additional file 6) had intensities distributed from 2.12-14.31. Approximately 59% of IT/Xl-PSIDs had average intensity values greater than four; most were in the 9^th^ and 10^th^ equal tally deciles (Figures 4E, 5A). When IT/Xl-PSIDs were grouped into equal intensity deciles, the 1^st^ and 4^th^ deciles contained the most IT/Xl-PSIDs (Figure 5B). IT/Xl-PSIDs with the highest intensities were mostly subunits for sodium/potassium/hydrogen transporting ATPases (Table 6D).

### **Putative transcription factors**

For this category of genes, 43.4% of the identified pTF/Xl-PSIDs have average intensity values above four (Figure 4F). In contrast to IET/Xl-PSIDs, DF/Xl-PSIDs, and IT/Xl-PSIDs intensity values distributions, the majority of pTF/Xl-PSIDs are in the 1^st^ equal tally decile (Figure 5A) as opposed to the 10^th^. However, when grouped into equal intensity deciles, the 1^st^ and 4^th^ deciles contained the most pTF/Xl-PSIDs (Figure 5B). The pTF/Xl-PSIDs with the highest intensities are listed in Table 6E.

### **Trends in Xl-PSID intensity distributions for inner ear gene categories**

We compared the distribution of Xl-PSID intensity values for the four gene categories in order to ascertain potential differences in the relative expression levels of inner ear genes based on functional classification. We observed that the pTF category, with the largest number of Xl-PSIDs (*n* = 795), was characterized by the lowest intensity value distribution of all the gene categories. Moreover, a larger percentage of the pTF/Xl-PSIDs (41.3%, see Additional file 7) have GCOS absent calls as compared with Xl-PSIDs in the other categories (20.3%-28.9%, see Additional files 3, 4, 5 and 6). We further noted that the largest proportions of DF/Xl-PSIDs (19/106), IT/Xl-PSIDs (21/128), and IET/Xl-PSIDs (86/361) were found in the 10^th^ equal tally decile (Figure 5A).

### **Manual curation efforts improved* X. laevis* GeneChip® annotation**

We implemented DAVID analysis to assess whether or not manual curation improved the annotation results for IET/Xl-PSIDs (*n* = 453), the largest category with manually-linked HGNC symbols. To this end, we compared the outcomes of DAVID analyses for IET/Xl-PSIDs to that of IET/HGNC symbols (see Additional file 8). The IET category of 453 IET/HGNCs was represented by 447 DAVID IDs, with one orphan IET/HGNC. DAVID grouped inner ear specific GO terms (“*inner ear development”, “inner ear morphogenesis”,* and *“sensory perception of sound”*) into two IET/HGNC functional annotation clusters. In contrast, for the corresponding 453 IET/Xl-PSIDs, no inner ear specific GO terms were recovered from analysis of the DAVID annotation clusters. Furthermore, the number of IDs that DAVID associated with IET/HGNCs (447) was greater than the number of IDs that DAVID associated with the IET/Xl-PSID counterparts (424). Moreover, the number of orphan IET/Xl-PSIDs (17) was greater than the single IET/HGNC orphan. Taken together, these findings suggest that manual curation of the *X. laevis* GeneChip® by assignment of HGNC symbols to the Xl-PSIDs improved annotation.

### **Identification of putative human inner ear orthologues in the* Xenopus tropicalis* genome**

**Figure 6:**
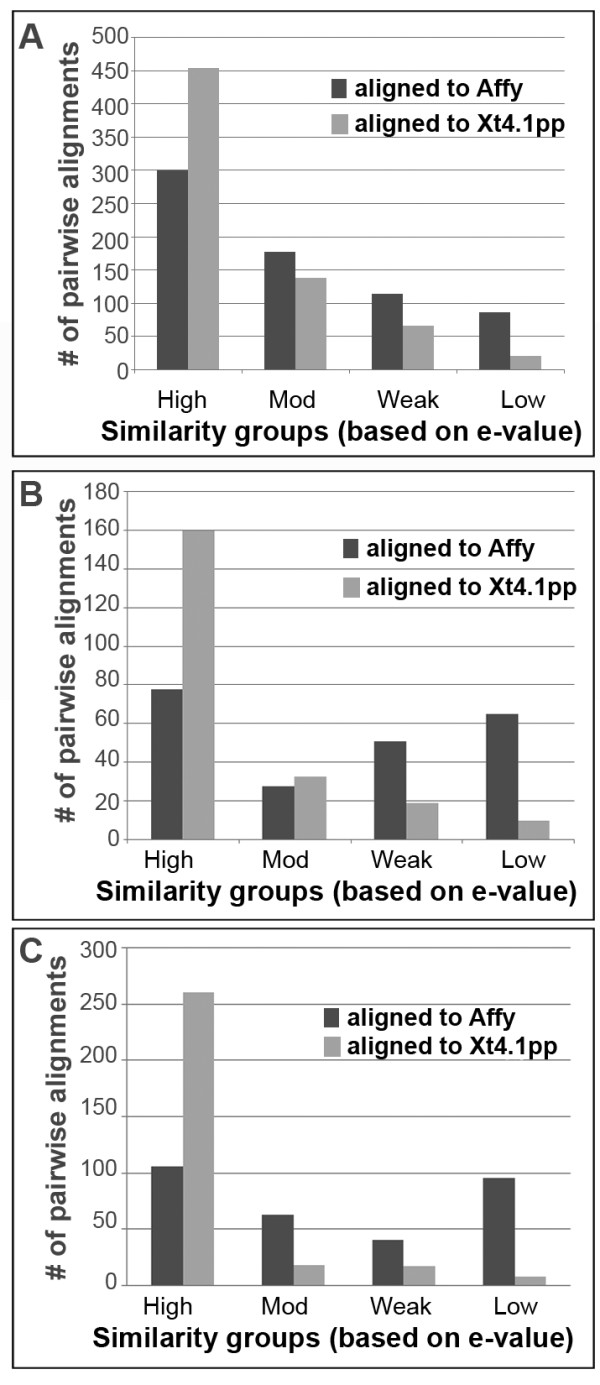
**BLAST analysis of gene category alignments** Histograms showing the number of protein sequences for genes in three inner ear categories that aligned to Xl-PSID consensus sequences (*Affy*) or *X. tropicalis* 4.1 predicted proteins (Xt4.1 pp) using BLAST algorithms: **A**, IET/HGNC (*n* = 681); **B**, DF/HGNC (*n* = 222); **C**, IC/HGNC (*n* = 306). Pairwise alignments were sorted into similarity groups based on e-value (high = 0-10^-100^; mod = 10^-99^ to 10^-50^; weak = 10^-49^ to 10^-15^; low = E > 10^-14^, data not shown).

In order to determine the extent to which *Xenopus* is a practical model organism for auditory and vestibular research, we used the products of our manual curation efforts to evaluate whether genes associated with inner ear function in other organisms (human, rat, mouse, and chicken) were present within the *X. tropicalis* genome. To this end, we used the BLASTP algorithm to determine whether HGNC human protein sequences from the IET, DF, and IC gene lists aligned with curated *X. tropicalis* 4.1 predicted proteins from the Joint Genome Institute (JGI) *X. tropicalis* sequencing project (Figure 6; see Methods). The average e-value for HGNC/Xt4.1 predicted protein mappings (IET, 0.01 + 0.17; DF, 0.02 + 0.18; IC, 0.01 + 0.13) were lower than the average e-value for HGNC/Xl-PSID mappings (IET, 0.10 + 0.59; DF, 0.23 + 0.78; IC, 0.20 + 0.72). Moreover, the number and percentage of affirmative pairwise alignments between HGNC human protein and *X. tropicalis* 4.1 predicted protein sequences (1039) exceeded the number of affirmative pairwise alignments between HGNC human protein sequences and Xl-PSIDs (855). These sequence similarity alignments demonstrate that more orthologues with high similarity to human proteins from all three gene lists were identified in the *Xenopus* genome than on the *X. laevis* GeneChip® (Figure 6).

## **Discussion**

### **Microarray technology for transcriptional profiling of inner ear endorgans**

Limited access to human inner ear RNA mandates the use of model organisms, such as *Xenopus laevis* for transcriptome analysis. Cellular and molecular investigations of the inner ear are challenged by the size and location of the organ. The human cochlea measures almost 1 cm in size while the entire *Xenopus* inner ear is about one third of that size; both are encased by the temporal bone, the densest bone in the body. We overcame the constraints imposed by the inaccessibility of the inner ear through implementation of rigorous surgical procedures that yielded high quality RNA from a small amount of inner ear tissue. Reproducibility between our arrayed biological replicates was evidenced by the similar intra-chip SDs for Xl-PSID intensity values and the low inter-chip SDs. These results illustrate the efficacy of our protocols in restricting biological and technical variation between replicates that may be introduced by experimental procedures such as tissue isolation, RNA extraction, and array hybridization.

The usefulness of inner ear genetics as an approach to develop treatments for inner ear disorders can be heightened through analysis of the relative expression of genes within the transcriptome. Data normalization is a critical step in achieving this objective. Box plots and MvA plots of normalized data showed low inter-chip variability as compared to plots constructed with pre-normalized data, demonstrating the efficacy of the normalization method. We elected to use data normalized with GCRMA because this data normalization method performs well with regard to accurate detection of low abundance transcripts and precision [62,63]. This data normalization method also is recommended when the objectives are to identify differentially expressed genes, or to estimate relative gene expression.

The ability to assess whether microarray intensity values are predictors of verifiable gene expression facilitates the interpretation of microarray data. In particular, it is useful to identify an intensity value, or threshold, above which PCR methods are likely to independently validate gene expression. Inner ear cDNA library clones represent expressed sequences and thus are a useful tool for assessing whether expressed sequences are detected in the array. The combination of GCOS absent calls and intensity levels of cDNA clones represented on the *X. laevis* GeneChip®, led us to predict that an Xl-PSID intensity value greater than or equal to four is likely to represent an expressed sequence that can be confirmed by RT-PCR. As expected, RT-PCR experiments with inner ear RNA confirmed the presence of 100% of eight genes that mapped to Xl-PSIDs with average intensity levels above six.

### **Annotation curation expands the biological relevance of the microarray data**

Transcriptome analysis is facilitated by genomic sequence data and quality gene annotation for the species of interest. The quality and the extent of annotation have been acknowledged as particular impediments to the advancement of transcriptomics [53,54,64-66]. For this reason, the National Human Genome Research Institute (NHGRI) initiated the Encyclopedia of DNA Elements (ENCODE), a project aimed at the functional annotation of all elements in the human genome [67].

The utility of the *X. laevis* GeneChip® is reduced by the number of arrayed genes (Xl-PSIDs) with no known biological function, a limitation that stems in part from the unsequenced *X. laevis* genome. We enhanced the biological relevance of the data by using a variety of computational strategies to link Xl-PSIDs to HGNC official gene symbols. The linkage of HGNC symbols to Xl-PSIDs exploited the detailed annotations of human genes as compared to other species. Sequence similarity mapping and semantic keyword querying facilitated the assignment of putative functions important for inner ear processes. Our *X. laevis* GeneChip® annotation efforts focused on five gene categories relevant for inner ear function: inner ear tissue (IET; *n* = 453), deafness (DF; *n* = 139), ion channels (IC; *n* = 74), ion transporters (IT, *n* = 180) and transcription factors (pTF, *n* = 795).

No single tool is sufficiently robust to assign function to genes from a species such as *X. laevis,* whose genome has not been sequenced. In order to impart biological function to our microarray data, it was mandatory to combine many curation approaches (Table 5, see Additional file 9). We found that the best approach involved combining reading the scientific research literature, keyword and nucleotide database queries, and functional annotation clustering with data-mining tools from the DAVID Bioinformatics Resource. DAVID was useful for providing information about groups of Xl-PSIDs through functional clustering. For example, DAVID analysis of the 10^th^ deciles (equal intensity, equal tally) revealed many GO categories associated with genes commonly found in all tissues and not exclusive to the inner ear, and identified the orphan Xl-PSIDs with no known annotation.

### **Gene groups facilitate the analysis of trends in the* X. laevis* inner ear transcriptome**

Through transcriptional profiling of the inner ear, we aimed to garner a comprehensive perspective of an understudied organ. The implementation of gene lists and decile groupings facilitated the analysis of the inner ear transcriptome by restricting our focus to a subset of Xl-PSIDs culled from known inner ear genes and from Xl-PSIDs with intensity values in the top 10%. The combination of these two approaches allowed us to identify patterns in the relative intensities of Xl-PSIDs, to compare *Xenopus* inner ear genes to the known inner ear genes of other species, and to gain insight about the contribution of genes with no known function to the inner ear transcriptome.

Trends in the intensity values for Xl-PSIDs were extricated through histogram and decile analysis. We observed that the distribution of Xl-PSID intensities in the histograms for categories with predicted inner ear function resembled the cumulative histogram for all Xl-PSIDs. We also noticed that all gene categories are represented in all equal tally and all equal intensity deciles (except for the 10^th^ equal intensity decile, where DF/Xl-PSIDs, IT/Xl-PSIDs and pTF/Xl-PSIDs were absent). Average intensity values were as follows: cumulative Xl-PSIDs, 5.63; IET/Xl-PSIDs, 6.46; DF/Xl-PSIDs, 6.04; IC/Xl-PSIDs, 5.32; IT/Xl-PSIDs, 5.55; pTF/Xl-PSIDs, 4.36.

We interpret the similarities between Xl-PSID distributions for the inner ear transcriptome and the gene categories as indicating that the gene categories are representative of the whole inner ear transcriptome. This outcome is interesting because the inner ear research that formed the basis for our selection of gene categories was rich in the science of mechanosensory hair cells whose numbers comprise very few cells of the inner ear. For example, the auditory hair cells in a juvenile *Xenopus* animal total approximately 3000 [68,69]; whereas, cochlear hair cells of the human inner ear number approximately 20,000 [5,7]. Nevertheless, the gene categories captured data that encompassed a full range of Xl-PSID intensity values.

The utility of *Xenopus* as a model organism for inner ear research is supported by the commonality we observed between the *X. laevis* transcriptome and the outcomes of gene analyses for other species traditionally used for auditory and vestibular research. Many of the common genes are ion channels and transporters, transcription factors, gap junction proteins, cytoskeletal proteins, and structural proteins that have been implicated in inner ear function in humans and mice [21,70-72].

Various genes common to the *X. laevis* inner ear and those of other species are associated with deafness, including structural proteins (collagen, type II, alpha 1 (*COL2A1*); collagen, type I, alpha 1 (*COL1A1*); and tectorin alpha (*TECTA*)), all of which have corresponding DF/Xl-PSID intensity levels above four. Of the 14 ion channel genes identified in both human cochlear and mouse organ of Corti cDNA libraries by Gabashvili et al. [66], nine were represented on the *X. laevis* GeneChip®. Moreover, seven of these nine genes corresponded to Xl-PSIDs with intensity values above four (potassium large conductance calcium-activated channel, subfamily M, alpha member 1, *KCNMA1*; chloride intracellular channel 4, *CLIC4*; chloride channel, voltage-sensitive 3, *CLCN3*; potassium channel tetramerisation domain containing 12, *KCTD12*; potassium channel, subfamily K, member 1, *KCNK1*; voltage-dependent anion channel 1, *VDAC1*). Ion transporters that play a role in K^+^ cycling and maintenance of endolymph in the cochlea of human, mouse, and rat [66,73,74] were also represented on the *X. laevis* GeneChip® by Xl-PSIDs with high intensities (ATPase, Na+/K + transporting, alpha 1 polypeptide *ATP1A1;* ATPase, Na+/K + transporting, beta 1 polypeptide, *ATP1B1*; ATPase, Na+/K + transporting, beta 2 polypeptide, *ATP1B2*; FXYD domain containing ion transport regulator 3, *FXYD3*; gap junction protein, beta 2, 26 kDa, *GJB2*). Additionally, transcription factors implicated in hair cell regeneration in the chicken inner ear, such as jun D proto-oncogene (*JUND*), CCAAT/enhancer binding protein C/EBP, gamma (*CEBPG*)*,* and paired box 2 (*PAX2*) [75] were identified as pTF/Xl-PSIDs with intensities above four in the *X. laevis* inner ear. The bone morphogenetic protein *BMP4*[76], which is important for cochlea and sensory organ development in mouse and chicken, was also detected in *Xenopus* (IET/Xl-PSID).

The prevalence of similar genes identified in both the human cochlea and *Xenopus* inner ear support the notion that physiological processes essential for inner ear function are shared between the two species. It was notable that Xl-PSIDs with intensities in the top 1% (*CLU;* peripheral myelin protein 22, *PMP22*; tumor protein, translationally-controlled 1, *TPT1*; secreted protein, acidic, cysteine-rich (osteonectin), *SPARC*; eukaryotic translation elongation factor 1 alpha 1, *EEF1A1*) correspond to the most abundant transcripts identified in a human fetal cochlear cDNA library (*SPARC, EEF1A1*, and *TPT1*; [22]). Clusterin (*CLU*, the IET/Xl-PSID with the highest intensity) was found in human perilymph with high protein concentrations [77]; currently the function of this glycoprotein in the inner ear is unknown. Taken together, the identification of Xl-PSIDs from all five gene categories with high intensity values supports the use of *X. laevis* to advance our understanding of the genes critical for inner ear function. Moreover, previously uncharacterized genes are now found to have a putative function in the *Xenopus* inner ear.

Focusing our attention on Xl-PSIDs with the highest intensity values uncovered the genes that are predominant in the juvenile *X. laevis* inner ear transcriptome. Our analysis of genes associated with the top 10 Xl-PSIDs (hemoglobin, ribosomal proteins, ferritin, similar to ubiquitin C, and 1 unknown sequence), as well as DAVID analysis of the 10^th^ decile, revealed that Xl-PSIDs with the highest intensity values in the *X. laevis* inner ear are linked to cellular maintenance functions, especially “housekeeping”. These cellular maintenance genes were represented in greater numbers in comparison to genes specific to inner ear function such as IET/Xl-PSIDs and DF/Xl-PSIDs (7.1% and 1.6%, respectively of the 10^th^ equal tally decile and the IET/Xl-PSIDs, 7.5% of the 10^th^ equal intensity tally decile). These findings are consistent with observations by other researchers who have noted that genes influential in other tissue types (and not directly related to hair cell mechanotransduction) are highly expressed in the inner ear [23,42,70].

Finally, our DAVID analysis of the *Xenopus* inner ear transcriptome revealed that 13% of the Xl-PSIDs in the 10^th^ equal tally decile are “orphans” and have no annotation. Analysis of the highest Xl-PSID intensity values highlighted the predominance of Xl-PSIDs without gene titles in the *Affymetrix* annotation file (Xenopus_laevis.na32.annot.csv [61]); 12% of the 100 most highly expressed Xl-PSIDs and 20.6% of the 1218 Xl-PSIDs in the 10^th^ equal tally decile fell into this category. Taken together, these results imply that the roles of many genes important for inner ear function have yet to be defined. As functional characterization of genomes expands through the use of interdisciplinary approaches and cross-species analysis, knowledge of the genetic elements essential to inner ear function and dysfunction is expected to increase.

## **Conclusions**

The genus *Xenopus* affords unique opportunities for inner ear research because of its utility as a developmental model for genetic investigations as well as the amphibian capacity for regeneration of mechanosensory hair cells and neural tissue. While amphibians have furthered our understanding of inner ear hair cell mechanotransduction and physiology, the inner ear transcriptome of amphibians is not comparably well-characterized. For this reason, we implemented microarray transcriptional profiling for large-scale analysis of the *X. laevis* inner ear transcriptome. We heightened the functional significance of our analysis by targeting groups of genes considered essential for inner ear function. We overcame challenges faced by investigators working with organisms with unsequenced genomes through informatics approaches that significantly enhanced the annotation of the *X. laevis* GeneChip®. Our results suggest that the *Xenopus* inner ear transcriptome comprises genes that share significant sequence similarity with genes associated with non-syndromic deafness in other species (human and mouse), as well as a high abundance of Xl-PSIDs with no known annotation (20.6% of the 10^th^ equal tally decile).

We propose that the aforementioned putative mammalian orthologues and unknown Xl-PSIDs identified in this study represent ideal targets for functional analysis through genetic approaches. Our findings provide a resource that can be used by the *Xenopus* community for shared research enterprises such as XenDB [48], Xenbase [49] and the recently established National *Xenopus* Resource at the Marine Biological Laboratory [78] that produces transgenic *Xenopus*. Taken together, our results support the implementation of *Xenopus* as a viable model for inner ear research, especially for investigation of hair cell regeneration, morphogenesis, and organogenesis.

## **Methods**

### ***Xenopus***

Juvenile *Xenopus laevis* were obtained from Nasco (Fort Atkinson, WI). Animals (*n* = 21) were approximately 1 month old with an average weight of 2.4 ± 1.0 g and an average length of 2.7 ± 0.3 cm. Animal husbandry and surgical procedures were approved by the *Institutional Animal Care and Use Committee* (IACUC) of New Mexico State University.

### **RNA Isolation and preparation of replicates for array analysis**

Inner ear RNA was isolated from three groups of 5–10 juvenile *X. laevis* according to established methods [79]. We use the term “*replicate*” to refer to one of these samples of pooled inner ear RNA (10–19 inner ears each). All RNA replicates (*n* = 3) were quantified on the Agilent Technologies 2100 Bioanalyzer. Electropherograms were reviewed with the 2100 expert software before and after labelling with the GeneChip® One-Cycle Target Labelling kit (*Affymetrix*). RNA integrity number (RIN) values for the RNA replicates ranged from 8.4 to 9.7 (see Additional file 10). Labelling and array procedures were optimized and standardized at the MIT BioMicro Center.

Labelled antisense cRNA was prepared from each RNA replicate using the GeneChip® One-Cycle Target Labelling kit following the manufacturer’s protocol (*Affymetrix*). Labelled antisense cRNA produced from one RNA replicate was then hybridized to one *X. laevis* GeneChip® microarray and scanned by the GeneChip Scanner 3000 7 G (*Affymetrix*). Therefore, each GeneChip® “*replicate array*” probed the transcriptome of inner ear RNA from a population of 5–10 animals. Throughout this paper we refer to all the PSIDs on the GeneChip® as Xl-PSIDs (*n* = 15,611). However, less than 1% of these Xl-PSIDs (*n* = 120) are control PSIDs for specific genes from several species.

### **Data preprocessing with GCRMA**

The original (raw) data in *X. laevis* GeneChip® CEL files acquired from three replicate arrays were preprocessed [80] using GeneChip robust multichip analysis (GCRMA, [63,81]) methods to produce a single log_2_ transformed measure for the intensity level of every Xl-PSID on each replicate array. Intensity values are reported in arbitrary units (a.u.) of fluorescence. The open source Bioconductor packages “*affy*” and “*gcrma*" [82] implemented in R [83] were used for GCRMA analysis. Throughout this paper, we refer to the Xl-PSID intensity values that were adjusted with these preprocessing procedures as normalized data. The original CEL files and normalized data were submitted to the NCBI Gene Expression Omnibus [GEO: GSE37767, GSM927627, GSM927628, GSM927629] archive.

### **Replicate array analysis**

The 120 *Affymetrix* controls (Xlc-PSIDs) in the dataset were not included in the analysis of *X. laevis* gene expression patterns (*n* = 15,491). Genes represented by multiple Xl-PSIDs on the *X. laevis* GeneChip® were verified for similar expression levels and the highest intensity values were used in the functional analysis of inner ear genes. Normalized and raw/pre-normalized intensity values were used to construct box and MvA plots with the Bioconductor package “affyPLM” [82] implemented in R. All histograms produced in R were graphed using normalized GCRMA data.

### **Xl-PSID intensity detection calls and decile groupings**

Detection calls for each Xl-PSID (present (P), marginal (M), and absent (A)) were assigned by the *Affymetrix* GeneChip® Operating Software (GCOS, [84]) for every *Xenopus* inner ear (XIE) replicate array. The software scored 12,177 Xl-PSIDs as either “*M*” or “*P*” in at least one replicate array and 3,314 Xl-PSIDs as “*A*” in all three replicate arrays. We partitioned the Xl-PSIDs into equal tally and equal intensity deciles based on average intensity values in order to facilitate data analysis. Xl-PSIDs scored as “*Absent*” in all three replicate arrays were removed from the decile group analysis. The remaining 12,177 Xl-PSIDs were divided into deciles. Xl-PSIDs with the lowest average intensities were grouped in the first decile while those with highest average intensity were grouped in the 10^th^ decile.

### **Identification of* Xenopus* genes with putative inner ear function on the* X. laevis* GeneChip®**

#### **Selection of categories for inner ear functional gene analysis**

Powers et al. [55] implemented manual and large-scale computational approaches to expand annotation of the *X. laevis* GeneChip® Xl-PSIDs by linkage to ion channel genes, HGNC symbols identified via UniGene cluster IDs, or Swiss-Prot proteins from multiple species (human, mouse, fly and worm). Similar manual approaches were used to link *X. laevis* GeneChip® Xl-PSIDs to five categories of genes with expected inner ear function: (1) inner ear tissue genes, IET; (2) genes implicated in human deafness, DF; (3) genes for ion channels, IC, (4) genes for ion transport, IT; and (5) genes for transcription factors, pTF (see Additional file 2). Throughout this paper, HGNC nomenclature (capitalized gene symbols) is used in reference to human orthologues with sequence homology to Xl-PSID consensus sequences, and lowercase gene symbols refer to *X. laevis* genes.

#### **Inner ear tissue genes (IET)**

A list of 681 human orthologues was compiled from inner ear gene expression studies (cDNA library, microarray) of human, mouse, rat, and chicken [22-27,42,85]. Due to differences in inner ear gene designations, we determined the universal gene HGNC symbol that represents each gene by using the UCSC Genome Browser (human NCBI36/hg18 assembly, [86]).

#### **Human deafness genes (DF)**

Genetic mutations can cause hearing impairment and in the most extreme case, deafness. The OMIM (Online Mendelian Inheritance in Man) database [87] was queried in 2012 with the term “*deafness*” to compile a list of genes with mutations associated with non-syndromic and syndromic deafness in humans. The OMIM query was filtered to retrieve genes with an official gene symbol as well as known sequences and/or phenotypes, resulting in a final list of 222 HGNC symbols.

#### **Ion channel genes (IC)**

The IC list includes 306 ion channel HGNC symbols for α – γ subunits, gap junction proteins, and hemi-channels. HGNC symbols for ion channel genes were identified as described above and with UniProt [88]. IC genes were compiled from three sources, the Ion Channel Database^BETA^[89], the IUPHAR database (International Union of Basic and Clinical Pharmacology [90]), and Gabashvili et al. [66].

#### **Ion transport genes (IT)**

We identified a master list of HGNC symbols that facilitate transmembrane ion transport. The IT master list of 368 genes is enriched for genes that code for ion channel (IC) proteins (*n* = 306). The IT list also includes genes identified by querying the *Affymetrix* annotation file (Xenopus_laevis.na25.annot.csv [61]) using keywords such as “*transporter*” and “*calcium*”. This procedure identified 62 ion transport genes, which were combined with the 306 IC genes. We noted that a single Xl-PSID could be annotated with more than one HGNC symbol. Consequently, the IT category of 370 genes was represented by 180 IT/Xl-PSIDs. Manual curation efforts as described in Powers et al. [55] ensured that all IT/Xl-PSIDs identified by keyword query of the *X. laevis* GeneChip® annotation file were linked to ion transport in primary literature or other online databases. Several ion transport genes were found to be represented by multiple Xl-PSIDs on the *X. laevis* GeneChip®.

#### **Transcription factors (pTF)**

A list of putative transcription factor genes arrayed on the *X. laevis* GeneChip® was compiled using the NetAffx™ analysis center [91], Xenbase and DAVID analysis of Xl-PSIDs. First, the output from the query term “*transcription factor*” in the NetAffx™ analysis center (linked to Xenopus_laevis.na25.annot.csv file) was displayed as an *Annotation list* and downloaded as a *.tsv file using the Export center feature on the website. The *Affymetrix* annotations corresponded to known transcription factors, growth factors important in cell proliferation, and several hypothetical proteins. The varied annotations corresponding to the transcription factor semantic keyword query output prompted the designation of Xl-PSIDs in this category as “putative” (pTF/Xl-PSIDs, *n* = 888) as well as our use of DAVID analysis to validate the biological function of pTF/Xl-PSIDs. DAVID linked 651 DAVID IDs to 836 pTF/Xl-PSIDs, and identified 52 orphans. The first annotation cluster (highest DAVID enrichment score) assigned the GO term “*regulation of transcription*” to 70.8% of Xl-PSIDs in this category. Merging the pTF list (888) with the results a keyword search in Xenbase for “transcription factor”, added additional pTF/Xl-PSIDs and eliminated the false positives, culling this category to 795 pTF/Xl-PSIDs.

### **Sequence similarity alignments of* Affymetrix* Xl-PSIDs**

Protein sequences from IET, DF, and IC gene lists were collected from Ensembl [92] with the Biomart data-mining tool as described in Powers et al. [55]. BLAST algorithms (standalone BLAST version 2.2.15; TBLASTN and BLASTP, [93]) were used to compare sequences from the gene lists to *X. laevis* GeneChip® Xl-PSID consensus sequences [61] and to predicted proteins from the *X. tropicalis* genome assembly (4.1; proteins.Xentr4.fasta.gz, Xt4.1 predicted proteins [94,95]). The best sequence match was evaluated for similarity to *X. laevis* GeneChip® Xl-PSIDs or *X. tropicalis* predicted proteins using the following e-value criteria: high (e = 0-10^-100^), H; moderate (e = 10^-99^ to 10^-50^), M; weak (e = 10^-49^ to 10^-15^), W; and low similarity (e > 10^-14^), L. The similarity groupings H, M and W were designated as affirmative pairwise alignments. If more than one human protein aligned to an Xl-PSID, the human protein with the lowest e-value and the highest number of aligned amino acids was used to map the Xl-PSID to a HGNC symbol. HGNC symbols were used in further analysis of Xl-PSID expression patterns (see Additional files 3, 4, and 5). The BLASTN algorithm (version 2.2.15, [93]) was also used to compare the *Xenopus* cDNA clone sequences to Xl-PSID consensus sequences. Sequence alignments were sorted into similarity groupings (H, M, W, or L) as described above in order to identify affirmative pairwise alignments.

### **DAVID functional annotation clustering of Xl-PSIDs with high intensities, IET/Xl-PSIDs and pTF/Xl-PSIDs**

DAVID Bioinformatics Resources 6.7 [58,59] has a functional annotation clustering tool that was used to impart functional significance to three groups of Xl-PSIDs: 1. most highly expressed Xl-PSIDs in the 10^th^ deciles (Table 4), 2. pTF/Xl-PSIDs identified using a keyword query in the NetAffx™ analysis center (*n* = 888; see Additional file 11) and, 3. HGNC symbols from the IET gene list that formed affirmative pairwise alignments with Xl-PSIDs (*n* = 453; see Additional file 8). DAVID identified orphan Xl-PSIDs (without gene annotations) and accounted for duplicate Xl-PSIDs per transcript by using a singular DAVID ID for each transcript.

### **Linkage of sequences from* Xenopus* inner ear cDNA phage library clones to* Affymetrix* Xl-PSIDs**

Clones were randomly selected and excised from two cDNA phage libraries constructed from inner ear RNA isolated from juvenile *X. laevis* (XE, *n* = 96) and juvenile *X. tropicalis* (TE, *n* = 101) as reported in Serrano et al. [57]. Plasmid DNA was isolated using either the QIAprep® Spin Miniprep Kit (Qiagen) or a modified alkaline lysis procedure [96]. Restriction enzyme digests and agarose gel electrophoresis were used to determine clone insert sizes (*n* =197; 0.2 – 2.5 kb). All cDNA clones were sequenced on the ABI PRISM® 3100 Genetic Analyzer using the BigDye® Terminator v3.1 Cycle Sequencing Kit protocol (Applied Biosystems). In Align IR, ABI sequence data were edited, aligned into contigs, and formatted as FASTA files that were mapped to the Xl-PSIDs using the BLASTN algorithm as described above. Sequence data were submitted to the NCBI Expressed Sequence Tags database [dbEST: JK841025 - JK841234] archive.

### **RT-PCR verification of genes expressed on the* X. laevis* GeneChip®**

The SMART™ RACE cDNA Amplication Kit (Clontech) was used to confirm that genes detected on the microarray could be amplified with RT-PCR from juvenile *X. laevis* inner ear template RNA [79]. Primers for the coding regions of *gata3, pfn2, six1, pmp*22*, clu, matn2, clcknb*, and *scnn1b* were designed from Xl-PSID consensus sequences (see Additional file 12). Negative controls for this experiment included both a “No RT” control (reactions with only template RNA and primers) and a “No cDNA” contamination control (reactions with primers and no RT product as template). Positive PCR products were purified with QIAquick PCR purification kit (Qiagen) and partial fragments were sequenced for gene verification on the ABI PRISM® 3100 Genetic Analyzer according to established procedures [79]. Sequence data were submitted to the NCBI GenBank archive [GenBank: JX033705, JX033706, JX033707, JX033708, JX033709, JX033710*,* JX033711, JX035911].

## **Authors' contributions**

TRP analyzed microarray data, reviewed the literature to identify genes isolated from inner ear endorgans, executed the sequence similarity mappings, manually linked HGNC symbols to Xl-PSIDs, participated in data analysis, prepared and edited figures, and drafted the manuscript. CTP carried out RNA isolation/preparation for microarray analysis and participated in figure preparation. SMV carried out computational analysis of microarray data, facilitated large-scale linkage of HGNC symbols to Xl-PSIDs, and participated in figure preparation. EES conceptualized the project and experimental design, coordinated the study, participated in figure preparation and data analysis, and drafted the manuscript. All authors read, revised and approved the final manuscript.

## Supplementary Material

Additional file 1 ***X. laevis* GeneChip® data used to construct table and figures.** Click here for file

Additional file 2 Inner ear tissue (IET), deafness (DF), ion channel (IC), ion transport (IT) HGNCs and transcription factor (pTF) gene symbols. Click here for file

Additional file 3 TBLASTN results of IET/Xl-PSID affirmative pairwise alignments. Click here for file

Additional file 4 TBLASTN results of DF/Xl-PSID affirmative pairwise alignments. Click here for file

Additional file 5 TBLASTN results of IC/Xl-PSID affirmative pairwise alignments. Click here for file

Additional file 6 IT/Xl-PSIDs identified through data mining and keyword query. Click here for file

Additional file 7 pTF/Xl-PSIDs compiled from NetAffx™ analysis center, DAVID analysis and Xenbase. Click here for file

Additional file 8 DAVID analysis of IET/HGNC symbols and IET/Xl-PSIDs. Click here for file

Additional file 9 Annotation enhancement reveals complexities in data interpretation. Click here for file

Additional file 10 **Agilent bioanalyzer analysis of RNA isolated from* X. laevis* inner ear tissue.** Click here for file

Additional file 11 DAVID analysis of putative transcription factors. Click here for file

Additional file 12 Primers and RT-PCR products. Click here for file
