# Supplementary material for: Probing the Xenopus laevis inner ear transcriptome for biological function
Source: BMC Genomics. 2012 Jun 8;13:225. doi: 10.1186/1471-2164-13-225 (PMC3532188; doi:10.1186/1471-2164-13-225)
Supplement: Additional file 9 — Annotation enhancement reveals complexities in data interpretation. [file 1471-2164-13-225-S9.docx]

**Additional file 9- Annotation enhancement reveals complexities in data interpretation**

While using different curation approaches, we came across the following situations:

1. Sequence similarity mapping of 770 human protein sequences from IET, DF and IC gene categories to Xl-PSID consensus sequences resulted in 577 affirmative pairwise alignments (Table 5, additional files 4-6) with multiple human protein sequences aligning with a single Xl-PSID (IET, 20%; DF, 13%; IC, 51%). For example:
2. An HGNC symbol linked to an Xl-PSID with a different annotation.
   1. Annotations for two proteins from the same gene family

(e.g. LRP2 linked to an Xl-PSID annotated as LRP6, Additional file 5)

- 1. Two different proteins that share a conserved sequence motif

(e.g. COCH (e-value = 1.09E-24) linked to an Xl-PSID annotated as MATN2, Additional files 4 and 5).

1. Semantic keyword querying was successful in identifying groups of Xl-PSIDs important in ion transport (IT). However, restricting semantic querying to the *Affymetrix* annotation file caused vague descriptors to be overlooked. This type of analysis is dependent on the quality of annotations, which are infrequently updated (e.g. ATP1A1 is present in Table 6A and missing in 6D).
2. The NetAffx™ tool is more complete (updated frequently) and searches GO terms along with gene annotations associated with Xl-PSIDs. While querying for transcription factors, the resulting output retrieved some false positives due to keyword choice (e.g. transcription factor will retrieve growth factors).
3. GeneChip® probe set design includes multiple Xl-PSIDs that recognize alternative transcripts for the same gene (e.g. Xl.8573.2.S1_a_at and Xl.8573.2.S1_x_at, Table 6D).
4. In a few instances, an HGNC symbol was not determined. This was due to an Xl-PSID consensus sequence with no sequence similarity to a human protein orthologue (e.g. Xl.18325.1.A1_at Table 6D).
