# Supplementary material for: Probing the Xenopus laevis inner ear transcriptome for biological function
Source: BMC Genomics. 2012 Jun 8;13:225. doi: 10.1186/1471-2164-13-225 (PMC3532188; doi:10.1186/1471-2164-13-225)
Supplement: Additional file 10 — Agilent bioanalyzer analysis of RNA isolated from X. laevis inner ear tissue. [file 1471-2164-13-225-S10.pdf]

A. XIE1 RNA

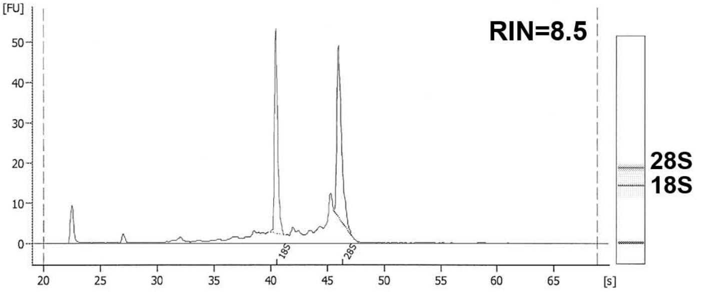

B1. XIE2 RNA 1

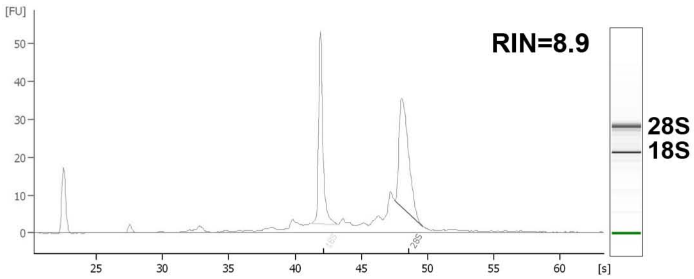

B2. XIE2 RNA 2

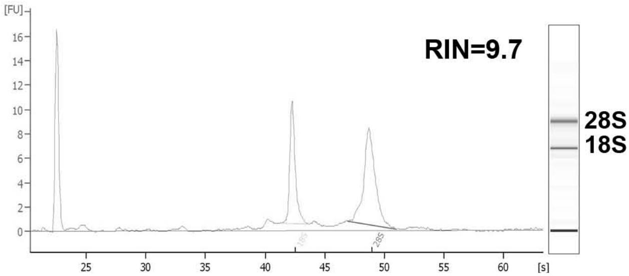

C. XIE3 RNA

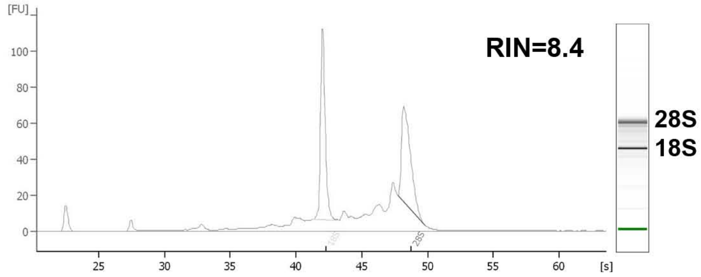

Additional file 10. Agilent Bioanalyzer analysis of RNA isolated from *X. laevis* inner ear tissue. Electropherogram and gels for RNA synthesized into cRNA and arrayed to XIE Chip 1(A), XIE Chip 2(B) and XIE Chip 3 (C). Note XIE Chip 2 was arrayed with cRNA synthesized from 2 pooled RNA samples. 28S rRNA and 18S rRNA peaks labeled. FU= fluorescence units RNA with RINs (RNA intregrity numbers) >8 were used in cRNA synthesis and labeling.
